# Hydrophobic Tag Protein Degraders: An Emerging Therapeutic Strategy for Cancer

**DOI:** 10.3390/ph19091477

**Published:** 2026-09-17

**Authors:** Tania Aguilera-Pineda, Siddhartha Kumar, Gargi Ghosal, Amarnath Natarajan, Adam R. Karpf

**Affiliations:** 1 Eppley Institute for Research in Cancer, University of Nebraska Medical Center, Omaha, NE 68198, USA; taniaandreaa.1998@gmail.com; 2 The Fred & Pamela Buffett Cancer Center, University of Nebraska Medical Center, Omaha, NE 68198, USA; gargi.ghosal@unmc.edu; 3 Division of Pharmaceutical Sciences, James L. Winkle College of Pharmacy, Department of Internal Medicine, College of Medicine, University of Cincinnati, Cincinnati, OH 45229, USA; kumar3sd@ucmail.uc.edu (S.K.); nataraah@ucmail.uc.edu (A.N.); 4 Department of Genetics, Cell Biology, and Anatomy, University of Nebraska Medical Center, Omaha, NE 68198, USA

**Keywords:** hydrophobic tag degraders, target protein degradation, mechanism of action, HyTD design, cancer therapy

## Abstract

Hydrophobic tag degraders (HyTDs) are a promising strategy for achieving targeted protein degradation (TPD). HyTDs are composed of a hydrophobic moiety conjugated via a linker to a ligand that binds to the protein of interest (POI). Binding induces a surface-exposed hydrophobic patch that mimics a misfolded protein, triggering degradation through the cell’s diverse quality-control networks, including the ubiquitin-proteasome system (UPS), ubiquitin-independent proteasome degradation (UbInPD), the autophagy-lysosome pathway (ALP), and the unfolded protein response (UPR). This distinct multi-pathway mechanism distinguishes HyTDs from traditional TPD strategies such as PROTACs and molecular glue degraders (MGDs), which rely on a narrow pool of specific E3 ligases. The effectiveness of HyTDs is influenced by the chemical properties of the hydrophobic moiety, the linker, and the ligand’s binding kinetics to the POI. To date, several relevant cancer targets, including the androgen receptor (AR), AKT serine/threonine kinase 3 (Akt3), and enhancer of zeste homolog 2 (EZH2), have been successfully targeted using HyTDs. Gaining deeper mechanistic insight into the nature of HyT degradation will be essential to further the potential of HyTDs as powerful tools in cancer research and therapeutics.

## 1. Introduction

Protein homeostasis, also known as proteostasis, refers to the network of cellular processes that regulate protein synthesis, folding, trafficking, and degradation [1]. Protein degradation plays a critical role in maintaining cellular homeostasis by preventing the accumulation of dysfunctional proteins, supporting continued protein production by removing misfolded or damaged proteins, and recycling amino acids [2,3,4]. In eukaryotic cells, damaged proteins or organelles can be cleared by two major pathways: the ubiquitin-proteasome system (UPS), which degrades short-lived, native, or misfolded proteins, and the autophagy-lysosome pathway (ALP), which targets long-lived proteins, organelles, and pathogens via endocytosis, phagocytosis, or autophagy [4,5,6]. While the UPS and ALP represent the primary pathways of protein clearance [7], they are not the only mechanisms available. Cells also leverage alternative degradation networks, including ubiquitin-independent proteasomal degradation (UbInPD) and the unfolded protein response (UPR), to manage misfolded or unfolded proteins.

**Figure 1:**
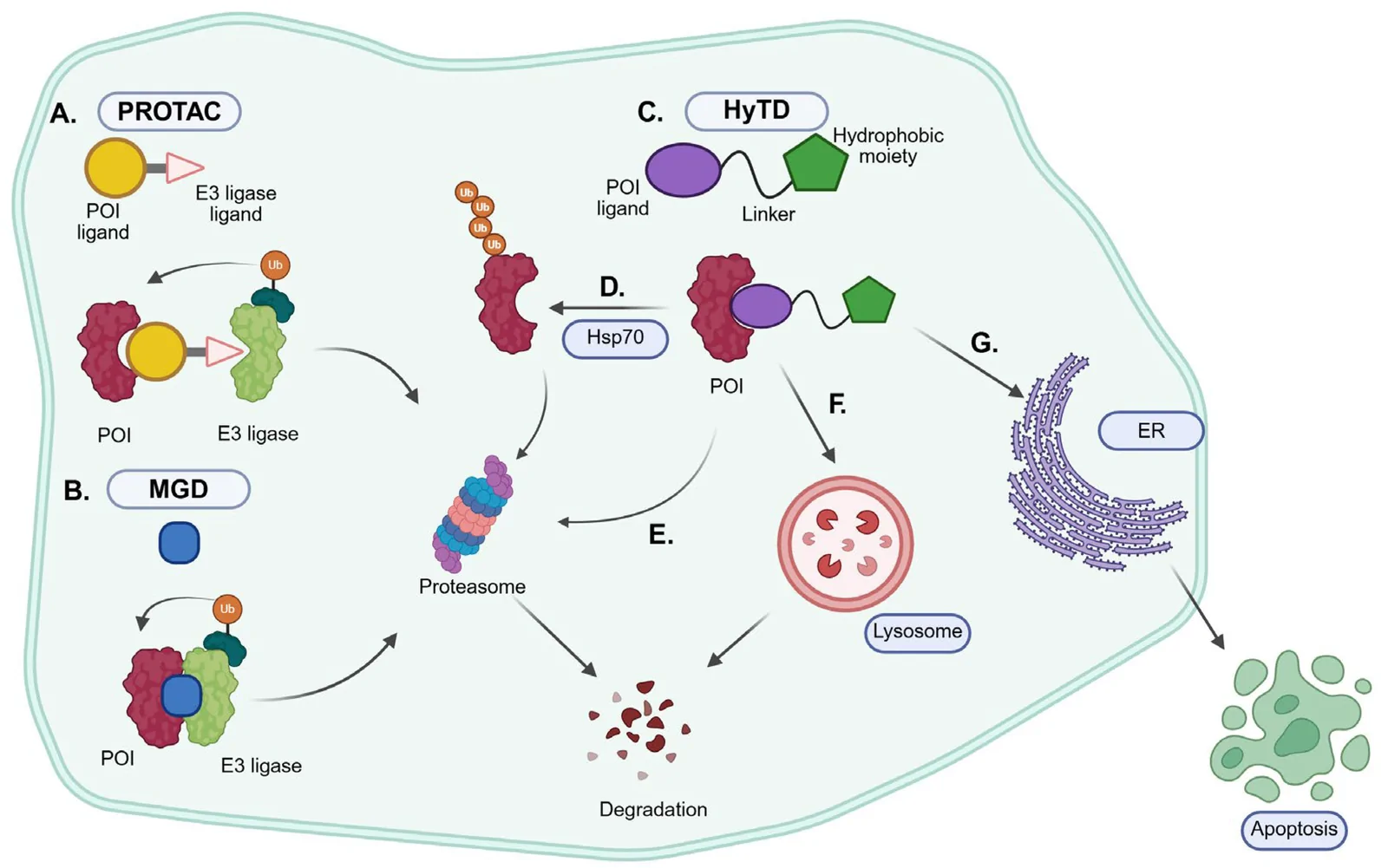
Schematic representation of targeted protein degradation mechanisms. (**A**) Degradation mechanism of PROTACs. PROTACs recruit an E3 ligase to the POI, forming a ternary complex that triggers POI ubiquitination and subsequent proteasomal degradation. (**B**) Degradation mechanism of MGDs. MGDs modify the surface of an E3 ligase to promote its interaction with the POI, thereby enabling ubiquitination and proteasomal degradation. (**C**) General structure of HyTDs. HyTDs comprise a hydrophobic moiety, a linker, and a ligand that binds to a POI. (**D**–**G**) Potential clearance pathways of HyTDs. (**D**) Chaperone-mediated UPS degradation. Binding of the HyTD to the POI recruits chaperone machinery to initiate UPS-mediated degradation. (**E**) Ubiquitin-independent protein degradation. The HyTD-bound POI is targeted directly to the proteasome for degradation without requiring ubiquitination. (**F**) Autophagy-lysosomal pathway degradation. The HyTD-bound POI is trafficked to and degraded within the lysosome. (**G**) The unfolded protein response clearance and apoptosis. Accumulation of the tagged POI at the ER triggers the UPR, ultimately culminating in apoptosis.

Endogenous protein degradation pathways have been harnessed by emerging therapeutic strategies known as targeted protein degradation (TPD), which aim to mediate the depletion of a POI [8]. Among TPD strategies, proteolysis-targeting chimeras (PROTACs) and molecular glue degraders (MGDs) have gained widespread attention. PROTACs are heterobifunctional molecules (700–1200 Da) composed of an E3 ligase ligand connected via a linker to a POI binding moiety. PROTACs bring the POI into proximity with an E3 ligase, forming a ternary complex that promotes POI ubiquitylation and degradation via the UPS (Figure 1A) [9,10,11,12]. MGDs are small monovalent compounds (300–500 Da) that reshape an E3 ligase receptor surface to chemically induce ternary complex formation with a target protein. This induced proximity drives the subsequent ubiquitination and degradation of the POI (Figure 1B) [11,13,14]. In contrast to PROTACs and MGDs, hydrophobic tag degraders (HyTDs) remain a less explored but valuable approach.

HyTDs are small, bifunctional molecules (400–650 Da) composed of a hydrophobic moiety, a linker, and a ligand that binds to a protein of interest (POI) (Figure 1C) [11,15,16]. This strategy is based on the principle that natively folded proteins typically conceal their hydrophobic regions within their core. However, abnormal exposure of these hydrophobic patches, commonly seen in unfolded or misfolded proteins, acts as a signal that triggers protein degradation [17]. HyTDs mimic protein misfolding signals, promoting their recognition and elimination by the chaperone-mediated protein quality control machinery [18]. HyTDs can direct otherwise stable proteins toward degradation by artificially mimicking this misfolded signal (Figure 1D–G). Importantly, compared to PROTACs and MGDs, HyTDs follow a more straightforward design strategy by combining a target-selective ligand with a hydrophobic moiety (the tag) to induce degradation [19]. Several studies have demonstrated the effectiveness of hydrophobic tagging in promoting the degradation of a range of protein targets, including HaloTag fusion proteins [16], glutathione-S-transferase (GST) [20], hepatitis B virus core (HBC) protein [21], androgen receptor (AR) [22], poly(ADP-ribose) polymerase 1 (PARP1) [23], steroid receptor coactivator-1 (SRC-1) [24], and anaplastic lymphoma kinase (ALK) [25], among others, demonstrating both target diversity and degradation capability.

HyTDs have been explored for their therapeutic relevance in cancer, particularly to avoid mechanisms of resistance often encountered with existing treatment approaches and to selectively degrade oncoproteins. This review explores the current understanding of HyTDs, examines the diverse mechanisms by which they induce target protein clearance, including the UPS, the ALP, the UbInPD, and the UPR, and explores their emerging utility in cancer therapy.

## 2. Hydrophobic Tag Degraders: Origin and Purpose

Targeting and eliminating oncogenic proteins offers a promising approach for treating cancer [26,27]. Traditionally, biologically active small molecule inhibitors (SMIs) have played a vital role in probing cellular mechanisms and impairing protein function for therapeutic benefit [28]. However, the majority of SMIs rely on occupancy-based inhibition, which limits their effectiveness to proteins with well-defined binding pockets [29]. In contrast, small-molecule-induced protein degradation has emerged as a powerful alternative strategy for targeting disease-related proteins, including those previously deemed “undruggable”. The protein degrader approach offers a broader therapeutic scope by shifting the focus from inhibiting a specific protein function to eliminating the protein entirely [30]. While PROTACs, MGDs, and HyTDs all drive target protein clearance, they have different mechanisms of action and are guided by distinct design principles. The primary strength of PROTACs is that, after degrading the POI, they can re-engage in degrading newly synthesized POI through a pseudo-catalytic mechanism [31]. However, their high molecular weight often leads to poor membrane permeability and unfavorable pharmacokinetics, and they are further limited by a narrow pool of E3 ligases and by the hook effect at high concentrations [9,19,32,33,34]. In contrast, MGDs have a lower molecular weight, better cellular absorption, and an innate ability to target proteins without a binding pocket, thereby avoiding the hook effect [35,36]. Yet, unlike PROTACs, MGD development is bottlenecked by a lack of rational design principles, leaving most discoveries dependent on serendipitous screening approaches [14].

**Figure 2:**
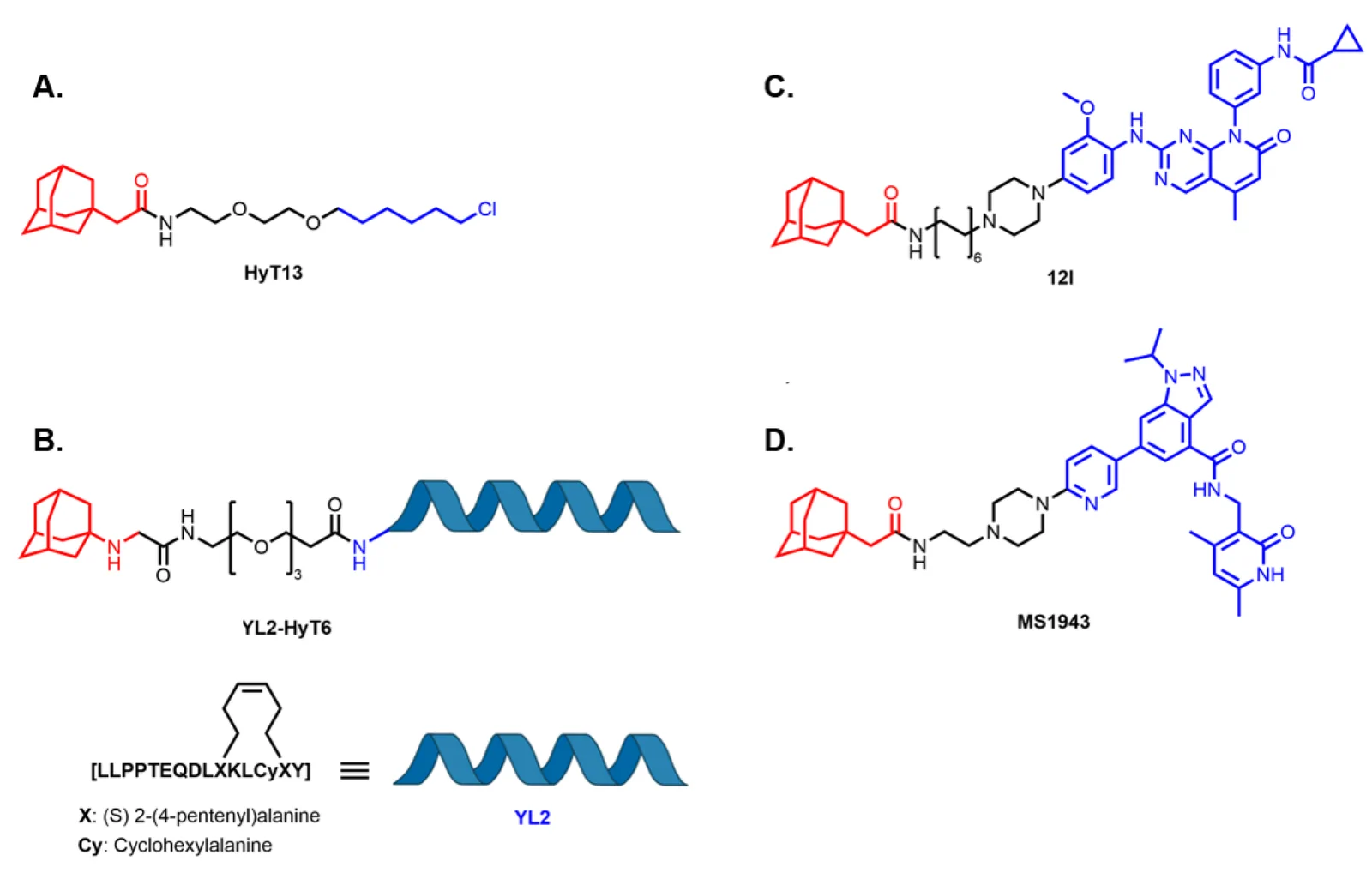
Chemical structures of representative HyTDs. The red structures represent the hydrophobic moiety; the black structures represent the linker, and the blue structures represent the POI ligand. (**A**) HyT13, targets HA-EGFP-HaloTag; (**B**) YL2-HyT6, targets SRC-1; (**C**) 12l, targets Akt3; (**D**) MS1943, targets EZH2.

HyTDs effectively bridge the gap between PROTACs and MGDs by combining the straightforward design rules of PROTACs with a lower overall molecular weight [16,19,37]. HyTD compounds work by exposing a hydrophobic moiety on the targeted POI; this exposed hydrophobicity serves as an essential signal to cellular quality-control systems, marking the POI as misfolded or damaged and routing it for degradation by the cellular quality control machineries [38,39]. The HyTD strategy was first implemented in 2011 through the development of the first reported HyT degrader, HyT13, which consists of an adamantane tag (hydrophobic moiety), a polyethylene glycol (PEG) linker, and a chloroalkane group for specific, irreversible binding to HaloTag protein, a modified bacterial haloalkane dehalogenase engineered for covalent conjugation [40] (Figure 2A). HyT13 successfully degraded the fusion protein hemagglutinin-EGFP-HaloTag (HA-EGFP-HaloTag), providing the first instance of HyT activity [16]. Building on these foundational in vitro models, HyTDs have demonstrated viable in vivo pharmacokinetics; notably, the adamantane-based EZH2 degrader MS1943 (Figure 2D) exhibited favorable pharmacokinetics in mice, demonstrating robust bioavailability across both intraperitoneal (i.p) and oral (p.o) administration routes [41].

## 3. Cellular Protein Degradation Systems

### 3.1. The Ubiquitin-Proteasome System

The ubiquitin-proteasome system (UPS) is the primary pathway responsible for the degradation of unfolded and misfolded proteins, which are toxic to the cell [42]. Misfolded proteins often expose hydrophobic regions that serve as degradation signals, triggering recognition and elimination by the UPS [17,43]. The UPS system is an elaborate framework composed of multiple molecular machineries that work together to maintain cellular homeostasis. It consists of enzymes and cofactors that modify protein substrates via ubiquitin, which is then degraded by the 26S proteasome [44].

Ubiquitin conjugation to target proteins is mediated by a multistep cascade reaction. First, ubiquitin-activating enzymes (E1) use ATP hydrolysis to form a thioester bond between the C-terminal glycine of ubiquitin and a cysteine residue in the E1’s active site. The activated ubiquitin is then transferred to a ubiquitin-conjugating enzyme (E2), forming a thioester bond. Finally, the ubiquitin-charged E2 enzymes interact with one of hundreds of ubiquitin ligases (E3), which transfer ubiquitin to the target protein [45,46]. The E3 ligase primarily determines substrate specificity by directly recognizing and binding the target protein [47,48]. After the transfer of ubiquitin to a lysine residue in the target protein, additional ubiquitin molecules can be assembled to form polyubiquitin chains through lysine residue (K48) linkage, which acts as a signal for degradation by the proteasome [45,49,50].

The binding of a POI by a HyTD mimics the structural features of unfolded or misfolded proteins, triggering recognition and degradation by the UPS [39]. Although the mechanism of action of HyTDs has not been fully elucidated, it is believed that HyTDs exploit the cell’s natural chaperon-substrate recognition cycle to initiate UPS-mediated degradation of target proteins. Molecular chaperones are proteins that form complexes with unfolded or misfolded proteins by trapping their exposed hydrophobic surfaces and assisting their folding or refolding. However, when proper folding cannot be achieved, or the accumulation of unfolded or misfolded proteins exceeds chaperone capacity, these proteins are directed toward UPS-mediated degradation [51,52,53]. Key chaperones and co-chaperones, such as Hsp70, Hsp40, CHIP, and BAG-1, may direct the HyTD-bound (i.e., tagged) POI toward degradation.

**Figure 3:**
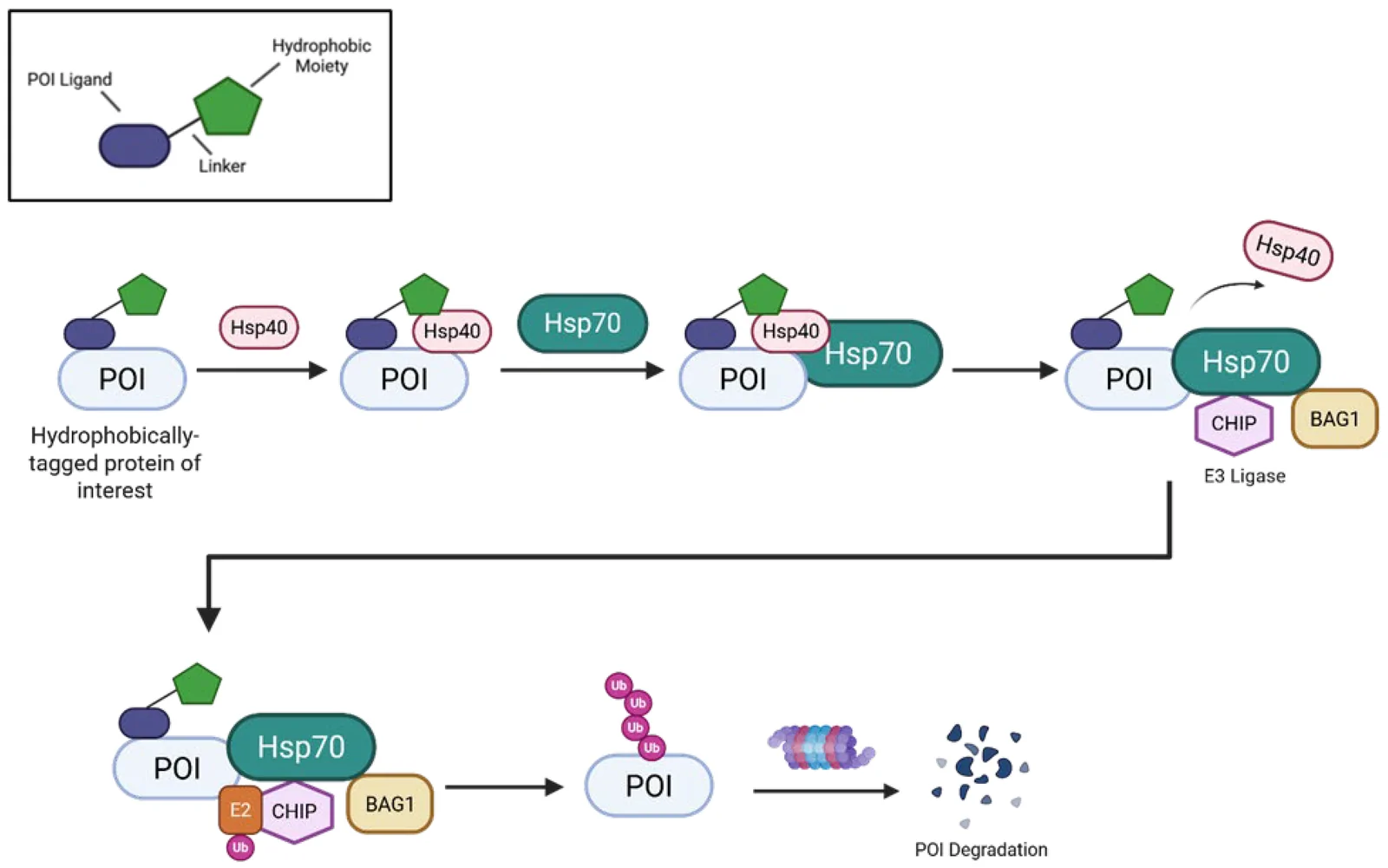
Hypothetical model for HyTD-mediated protein degradation by the UPS. The top schematic shows the general structure of HyTDs. Hsp40 initially recognizes the hydrophobically tagged POI and forms a complex with Hsp70, initiating a regulated chaperone cycle. Because the HyTD mimics protein misfolding, the POI stays in complex with Hsp70, increasing CHIP recruitment and subsequent polyubiquitination. BAG-1, in turn, promotes POI release from the complex and delivery to the proteasome, leading to proteasomal degradation.

In Figure 3, we present a hypothetical model for HyTD-mediated protein degradation. Initially, a co-chaperone in the Hsp40 (DnaJ) family interacts with the tagged POI; the chaperone utilizes a specialized binding pocket to directly recognize the exposed synthetic hydrophobic tag [54]. Following initial recognition, Hsp40 recruits Hsp70, a major chaperone family present in both the cytosol and nucleus, to the complex, initiating a regulated cycle of protein processing [55]. Upon interaction with the POI and Hsp40, Hsp70 undergoes a conformational shift that stabilizes the protein interaction. Under normal conditions, this cycle facilitates native protein refolding [56]. However, because the HyTD is bound to the POI, it cannot be internalized into the protein’s hydrophobic core during folding attempts; it remains constantly exposed on the POI’s surface. This continuous exposure makes the POI look misfolded, trapping it in a repetitive cycle with Hsp40 and Hsp70 [57,58,59]. This prolonged interaction with the chaperone complex increases the likelihood of recognition by CHIP, a bifunctional enzyme that serves as both an E3 ubiquitin ligase and a binding partner for Hsp70 and Hsp90, facilitating the ubiquitination and subsequent degradation of chaperone-bound substrates [60,61]. In a stochastic process, CHIP, which is more commonly associated with Hsp70 than Hsp90, binds to Hsp70–substrate complexes and recruits an E2 ubiquitin conjugation enzyme to tag the substrate for degradation [62].

Although CHIP plays a central role in marking chaperone-substrates for degradation, it does not act alone. It cooperates with the co-chaperone BAG-1, which acts as a nucleotide exchange factor (NEF) and couples Hsp70 to the proteasome, facilitating the efficient delivery of ubiquitinated substrates [63]. BAG-1 interacts with Hsp70, modulates its activity, and promotes protein release from the Hsp70 chaperone [64,65]. CHIP and BAG-1 thus work together to redirect Hsp70 activity from protein folding to protein degradation. During the sorting of chaperone-bound proteins to the proteasome, CHIP mediates their polyubiquitination, which is the rate-limiting step for proteasomal degradation. BAG-1 does not bypass this requirement; as a NEF, BAG-1 binds Hsp70 and facilitates the disassociation of the ubiquitinated POI. Concurrently, BAG-1 directs the ubiquitinated POI towards the proteasome [63]. The association of CHIP and BAG-1 with Hsp70 effectively converts these molecular chaperones into components of the degradation machinery.

Hsp90 is a highly conserved molecular chaperone found in multiple cellular compartments, including the endoplasmic reticulum (ER) and mitochondria [66]. It plays a key role in maintaining the stability and functional integrity of regulatory proteins, including receptors, protein kinases, and transcription factors [67]. Hsp70 and Hsp90 work in tandem across a wide range of cellular processes, forming a sequential and cooperative chaperone cascade. In this system, Hsp90 functions downstream of Hsp70, enhancing protein folding and, together with other chaperones and co-chaperones, supporting the maturation of regulatory proteins [68,69]. Although Hsp90 has not been directly implicated in the hydrophobic tag-mediated degradation, it remains a valuable tool for studying HyTDs. Choi et al. [24] used 17-AAg, a well characterized Hsp90 inhibitor that promotes the upregulation of Hsp70 expression. Upon cellular treatment with 17-AAg, Hsp70 levels increased, as did the activity of YL2-HyT6, a HyTD in which the YL2 ligand targets the steroid receptor coactivator-1 (SRC-1) (Figure 2B). These data lead to the hypothesis that Hsp90 inhibitors may enhance the action of HyTDs, suggesting future combination treatment strategies.

### 3.2. The Ubiquitin-Independent Proteasome System

The UPS represents the major route by which the cell degrades misfolded or unfolded proteins [70,71]. Canonically, the 26S proteasome complex is composed of two subcomplexes: a catalytic core particle (20S proteasome) and two terminal 19S regulatory particles that recognize polyubiquitinated substrates and translocate them into the 20S chamber for degradation [72,73]. However, a subset of eukaryotic proteins undergoes ubiquitin-independent proteasomal degradation (UbInPD) [70]. Specifically, native proteins containing large unstructured segments, referred to as intrinsically disordered regions (IDRs), or proteins with entirely disordered sequences (intrinsically disordered proteins, IDPs), are highly susceptible to direct degradation by the 20S proteasome [74,75]. Well-established endogenous substrates that are cleared by UbInPD include ornithine decarboxylase (ODC) [76], thymidylate synthase (TS) [77], p53 [78], p21 [79], and C-Fos [80].

The intrinsic capability of the proteasome to degrade substrates without polyubiquitin chains was mirrored in the development of HyTDs. Specifically, Shi et al. [81] discovered that target protein degradation can be driven by a POI ligand linked to a tert-butyl carbamate-protected arginine (Boc_3_Arg) moiety. Utilizing glutathione-S-transferase α1 (GST-α1)—a prominent target for anticancer chemotherapy [82]—as a POI, Long et al. [20] developed the HyTD EA-Boc_3_Arg. In HeLa and NIH 3T3 cells, this molecule induced the degradation of GST-α1. Crucially, no protein degradation occurred upon treatment with unmodified EA or isolated linker controls, demonstrating that the Boc_3_Arg moiety specifically targets the POI for degradation. To test that this degradation strategy was widely applicable and not unique to the EA scaffold, a second HyTD was evaluated: Fur-Boc_3_Arg. This molecule replaced the EA ligand with a structurally distinct thiobenzofurazan (Fur) GST-α1 ligand while retaining the Boc_3_Arg moiety. When incubated in cell lysates, Fur-Boc_3_Arg degraded GST-α1 with similar efficiency as EA-Boc_3_Arg. Mechanistic evaluations revealed that while proteasome inhibitors blocked Boc_3_Arg-mediated POI degradation, no corresponding accumulation of ubiquitin occurred. Furthermore, omitting the ATP-regenerating system required for ubiquitylation and 26S proteasome-mediated degradation of ubiquitylated proteins did not inhibit POI degradation. Collectively, these observations demonstrate that the Boc_3_Arg moiety induces degradation of the POI by a mechanism that requires the proteasome but not ubiquitylation.

### 3.3. The Autophagy-Lysosome Pathway

The autophagy-lysosome pathway (ALP) is key in identifying and eliminating large, potentially harmful cellular structures, including protein aggregates and damaged or excess organelles. It is recognized as a vital adaptive response to diverse cellular stress conditions [83,84]. Various stresses activate autophagy, including nutrient deprivation, ER stress, and microbial infection [85]. ULK1, a serine/threonine kinase, acts as the primary initiator of the autophagy machinery and is regulated by phosphorylation by AMPK and mTORC1, thereby linking cellular energy status and nutrient availability to autophagy activation [86].

There are three autophagy pathways: macroautophagy, microautophagy, and chaperone-mediated autophagy (CMA). While all of these pathways share the ability to transport cytosolic material to lysosomes for degradation, they differ in the mechanisms by which the cargo is targeted to the lytic compartment [83]. The most prevalent form, macroautophagy, involves the formation of cytosolic double-membrane vesicles called autophagosomes, which form independently of the lysosomal membrane to sequester portions of the cytoplasm. These autophagosomes then fuse with lysosomes, releasing their inner contents into the lumen for degradation and recycling of cellular components [87]. During microautophagy, the lysosomal membrane undergoes random inward folding, forming an autophagic tube that engulfs cytosolic material. Vesicles develop at the top of the tube, fuse homotypically, and subsequently bud into the lysosomal lumen for degradation [88]. In CMA, individual proteins are selectively recognized, via a conserved pentapeptide motif, by a cytosolic chaperone, which escorts them to the surface of the lysosomes. There, the proteins are unfolded and translocated across the lysosomal membrane for degradation [89].

The hepatitis B virus core protein (HBC) serves as the primary structural building block of the viral nucleocapsid, the site of viral DNA reverse transcription and replication, and plays a critical role in mediating virus–host interactions that sustain chronic hepatitis B virus (HBV) infection [90]. To target this therapeutic vulnerability, Xu et al. [21] synthesized a library of HyTDs by conjugating the anti-HBV core ligand SP-5 to an adamantane hydrophobic moiety via variable-length carbon or PEG-based linkers. Structure–activity relationship (SAR) analysis identified HyT-S7, featuring a PEG-based linker, as the lead compound, demonstrating robust inhibitory activity and a marked capacity to drive selective, time- and concentration-dependent HBC degradation.

Comprehensive mechanistic evaluations revealed that co-treatment with the proteasome inhibitors MG-132 or bortezomib failed to rescue HBC levels, and 17-AAG similarly showed no impact on degradation kinetics. Instead, co-treatment with 3-methyladenine (3-MA), which blocks early-stage autophagosome formation, or chloroquine (CQ), which prevents late-stage lysosomal acidification, completely reversed HyT-S7-induced HBC degradation. Furthermore, the investigators observed significant expression shifts in key regulatory proteins of the macroautophagy machinery [91,92,93,94,95], including SQSTM1 (p62), LC3B, JUN, WIPI1, TMEM39A and TMEM41B. Collectively, these findings establish HyT-S7 as a pioneering antiviral HyTD that induces HBC degradation via the ALP.

### 3.4. The Unfolded Protein Response Pathway

The Unfolded Protein Response (UPR) is a critical cellular quality-control mechanism that maintains protein homeostasis in the ER [96,97]. Triggered by an accumulation of unfolded or misfolded proteins, the UPR initially works to restore homeostatic balance by mitigating the unfolded protein load, enhancing ER folding capacity, and eliminating slowly folding substrates [96,98]. These initial homeostatic efforts reduce the burden on the secretory pathway; however, sustained UPR signaling under chronic ER stress ultimately induces cell death [99,100].

Mechanistically, UPR signaling is driven by three main ER transmembrane-associated sensor proteins: inositol-requiring enzyme 1α (IRE1α), protein kinase R-like endoplasmic reticulum kinase (PERK), and activating transcription factor 6 (ATF6), whose activation is suggested to be upstream-regulated by the ER chaperone BiP [101,102]. Once activated, each sensor initiates a distinct signaling cascade to alleviate ER stress. PERK phosphorylates eIF2α, which limits protein synthesis [98,102,103]. Concurrently, the IRE1 pathway stimulates its intrinsic endoribonuclease activity to mediate the spliceosome-independent splicing of *XBP1* mRNA [97,98]. This splicing event generates a potent transcriptional activator that selectively upregulates downstream UPR target genes, including molecular chaperones that help clear the burden of misfolded proteins [102,104]. Finally, ATF6 is translocated to the Golgi apparatus and proteolytically cleaved, liberating an NH2 domain fragment that migrates to the nucleus to modulate gene expression networks required for ER recovery [98,105].

HyTDs exploit the UPR pathway to drive POI towards degradation. For example, Go et al. [23] developed a small-molecule HyTD targeting PARP1 by linking an intermediate of the clinical PARP inhibitor olaparib to a bulky, lipophilic fluorene moiety. This HyTD, named 3a, effectively induced selective PARP1 degradation in MDA-MB-231 triple-negative breast cancer (TNBC) cells. Mechanistically, this degradation triggered apoptotic signaling, as evidenced by upregulation of pro-apoptotic genes and markers (Bax, p21, and cytochrome c) and a decrease in the anti-apoptotic protein Bcl-2. Consequently, compound 3a demonstrated greater anticancer efficacy than olaparib alone, inhibiting TNBC cell growth by approximately 80% after 96 h of treatment at a concentration of 5 μM. Crucially, this clearance phenotype was accompanied by increased levels of XBP1s and the pro-apoptotic transcription factor CHOP [106]. This upregulation demonstrates that compound 3a-mediated target degradation engages the UPR network to drive selective ER stress-induced apoptosis in cancer models.

## 4. Design Principles for Hydrophobic Tag Degraders

The efficacy of HyTDs for inducing target protein degradation depends on several key factors, including the hydrophobicity and structure of the hydrophobic tag, the length and flexibility of the linker, the binding affinity of the POI ligand, and the pharmacological properties of the HyTD, including its membrane permeability.

### 4.1. Hydrophobic Moiety

The choice of hydrophobic moiety is critical, as ideal candidates must (1) maximize hydrophobicity to mimic misfolded proteins and promote recognition by cellular degradation machinery, (2) minimize molecular weight to preserve cell permeability, (3) comprise chemically diverse and commercially available scaffolds, and (4) maintain the structural integrity of the parent POI ligand to ensure that protein recognition and binding are not compromised [24,39,107]. A range of hydrophobic tags has been explored, including adamantane [22], norbornene [25], carborane [108], fluorene [23], tert-butyl carbamate-protected arginine (Boc_3_Arg) [81], pyrene [109], and menthoxyacetyl [110]. The adamantane group is the most extensively utilized due to its high chemical stability and favorable cell membrane permeability. Go et al. [23] fused various hydrophobic moieties, including adamantane, to the naked piperidine amine moiety of the PARP inhibitor olaparib. Despite its promising physicochemical properties, HyTD with an adamantane moiety exhibited limited ability to induce PARP degradation. In contrast, HyTDs incorporating a fluorene moiety demonstrated significantly higher degradation efficacy. Furthermore, Xie et al. [25] developed hydrophobic-tagged anaplastic lymphoma kinase (ALK) degraders by attaching various hydrophobic moieties, including adamantane, fluorene, Boc_3_Arg, oleic acid, and norbornene. While several of these compounds effectively induced protein degradation, those bearing a norbornene moiety were more potent. Additionally, modifying the chemical structure of the moieties can significantly influence HyTD potency. For instance, incorporating branched hydrophobic groups at the α-carbon adjacent to the peptide bond carbonyl linking the moiety to the linker has been associated with enhanced degradation activity [39]. Overall, strategically modifying hydrophobic moieties is a powerful approach to optimizing degradation.

### 4.2. Linker

Linker design is pivotal in determining HyTD efficacy, as hydrophobic tagging, analogous to other protein degrader approaches such as PROTACs, is highly dependent on linker composition. SAR studies are useful for evaluating the impact of linker length, composition, rigidity, and linkage point on the degrader activity of the POI ligand [111]. As with PROTACs, finding the optimal linker design is essential, and various flexible linkers—often derived from PEG or alkyl chains—are commercially available and have been incorporated into HyTDs to facilitate the synthesis of potent HyTag degraders across diverse targets [112].

For instance, Tae et al. [39] evaluated a series of HyTDs that shared the same triphenylmethyl hydrophobic moiety but differed in linker length. The authors found that as the number of atoms in the linker increased, the maximum degradation decreased, from 76% for the shortest linker to 28% for the longest. However, further shortening did not improve degradation potency. In a separate study, constructs lacking a linker, where the hydrophobic moiety was directly attached to the POI ligand, showed no degradation [110]. Consistent with this finding, Guo et al. [113] demonstrated that the linker length influenced HyTD efficacy. Specifically, longer, more flexible linkers were associated with reduced POI-binding affinity. Their results showed that while a one-carbon spacer improved binding affinity compared to a zero-carbon linkage, further expansion to three or more carbon spacers decreased binding affinity. These data suggest that while a minimal spacer is required, a longer linker length can reduce binding affinity and degradation efficiency.

Once an optimal linker length is identified, replacing it with a rigid linker, such as a phenyl [113] or ethynyl [114] group, can further improve aqueous solubility, cell permeability, and the pharmacokinetic properties of the degrader [112]. For example, incorporating a six-membered ring to increase linker rigidity in an SNS032-based CDK9-cyclin T1 degrader enhanced degradation potency compared to longer, more flexible linkers [110]. This highlights the need to balance linker rigidity and length to promote effective HyTD-mediated protein degradation.

### 4.3. Ligand Binding Affinity

An important factor influencing HyTD performance is the ligand-binding affinity to the POI. Structure-guided approaches are often used to improve the binding affinity of the designed POI ligand [115]. Binding kinetics are critical for HyTD efficacy, specifically the off-rate, which measures how quickly the ligand dissociates from the POI [116]. A slower off-rate ensures the HyTD remains on the target long enough to trigger the cellular quality-control machinery. Because of this, converting to covalent ligands is an optimal strategy to permanently anchor the tag to the target [116,117,118]. However, covalent engagement of the POI will eliminate HyTD’s pseudo-catalytic activity, thereby converting it into a single-turnover degrader [9].

### 4.4. Ligand-Assisted Covalent Hydrophobic Tagging

The human genome codes for over 20,000 proteins, yet only a fraction are considered druggable. Many target proteins are deemed ‘undruggable’ due to flat functional surfaces and the absence of well-defined ligand-binding pockets, posing significant challenges for conventional drug design strategies [119,120]. Among these are DNA and RNA binding proteins (DBPs and RBPs, respectively), which, despite their critical role in controlling or regulating gene expression, often lack defined binding pockets for small-molecule ligands [121,122,123,124,125]. To address this limitation, innovative strategies are needed to expand the utility of hydrophobic tagging for targeting these traditionally ‘undruggable’ proteins.

To overcome this hurdle, Wang et al. [117] reported a novel ligand-assisted covalent hydrophobic tagging (LACHT) strategy for target protein degradation of DBPs and RBPs. LACHT functions by utilizing a reactive hydrophobic tag that leverages a transient, non-covalent binding event to covalently attach the hydrophobic moiety to a lysine residue proximal to the initial binding site. Specifically, an N-Acyl-N-alkyl Sulfonamide (NASA) was employed as a cleavable linker to conjugate a non-covalent POI ligand with a hydrophobic moiety. NASA forms a covalent bond between the hydrophobic moiety and the protein, even when the ligand has low or moderate affinity. To confirm the practicality of this approach, BRD4, a bromodomain-containing protein from the BET family, was selected as a well-characterized model system for validation. A series of small-molecule degraders (SMDs) was synthesized by conjugating adamantane to JQ1, a noncovalent small-molecule ligand against the POI. Notably, SMD1 successfully tethers the adamantane moiety directly onto BRD4 via a chemically stable amide bond. After confirming this cellular target engagement, the tagged BRD4 was successfully routed for degradation. This validation provides a strong foundation for expanding the strategy to target proteins when only low-affinity surface ligands are available [126].

## 5. Hydrophobic Tag Degraders in Cancer Therapy

Targeted protein degradation (TPD) has emerged as a transformative strategy in cancer research and therapeutics, offering distinct advantages over traditional occupancy-based inhibitors in terms of dosing, side effects, drug resistance, and the ability to modulate “undruggable” targets [127,128,129]. However, many cancer-related proteins remain “undruggable” because they lack suitable ligand-binding pockets, rely on flat protein–protein interactions, possess highly conserved active sites among protein family members, or exhibit variable tertiary docking structures [130]. Among the diverse TPD platforms, HyTDs represent a unique class of molecules that may significantly advance cancer therapy.

Oncoproteins drive tumorigenesis by disrupting signaling pathways that regulate cellular growth, proliferation, and apoptosis [131]. These proteins are ideal therapeutic targets given their central role in cancer. Although the field is in nascent stages, HyTDs have now been engineered to effectively degrade multiple oncoprotein targets, including the androgen receptor (AR), enhancer of zeste homolog 2 (EZH2), steroid receptor coactivator-1 (SRC-1), phosphodiesterase 6δ (PDEδ), AKT serine/threonine kinase 3 (Akt3), anaplastic lymphoma kinase (ALK), and bromodomain-containing protein 4 (BRD4) (Table 1). Below, we discuss in detail HyTDs that have been successfully employed against three key oncoprotein targets.

### 5.1. Androgen Receptor

Androgen Receptor (AR) is a ligand-dependent nuclear transcription factor that mediates the actions of androgens such as testosterone and dihydrotestosterone and plays a pivotal role in the development and maintenance of the reproductive, musculoskeletal, cardiovascular, immune, neural, and hematopoietic systems [134]. AR is implicated in the incidence and progression of prostate cancer, which is the second most common cancer type among men globally [135]. Prostate cancer is an androgen-dependent tumor; thus, conventional treatment strategies focus on inhibition of the AR by utilizing androgen deprivation therapy (ADT) or second-generation AR antagonists, such as enzalutamide and apalutamide [136]. Despite the initial effectiveness of these treatments, patients eventually acquire resistance and progress to a more lethal stage, castration-resistant prostate cancer (CRPC) [137,138]. Recognizing that AR overexpression is a primary driver of CRPC development, Gustafson et al. [22] sought to bypass traditional inhibition by inducing AR degradation. To this end, they synthesized a series of novel AR HyTDs utilizing the high-affinity AR agonist RU590632 as the POI ligand, conjugated to an adamantane group via a PEG linker. The resulting lead compounds, SARD279, which features an 8-atom PEG with an ester linkage, and SARD033, which utilizes a longer PEG with an ether linkage, both maintained robust AR binding. These compounds achieved almost complete AR depletion at 10 μM in LNCaP cells, with DC_50_ values of 1 and 2 μM, respectively. The success of these early HyTDs paved the way for other delivery platforms designed to optimize solubility and target engagement. In a 2025 study, Lee et al. [133] developed a nanoparticle-based platform in which the AR ligand is attached to a hydrophobic polylactic acid (PLA) polymer as the hydrophobic moiety. To make the system soluble for delivery, they added a protective outer PEG shell that disassembles upon entering the cell. The resulting ARL-PLA compound effectively reduced AR expression in a concentration-dependent manner with a DC_50_ of 18.25 μM. Collectively, these studies demonstrate that hydrophobic tagging is a promising strategy for the targeted depletion of the AR.

### 5.2. AKT Serine/Threonine Kinase 3

Akt is a serine/threonine protein kinase that regulates many cellular functions, including cell proliferation, survival, apoptosis, and metabolism. Its aberrant activation is a hallmark of numerous human cancers, including non-small cell lung cancer (NSCLC) and head and neck squamous cell carcinoma (HNSCC) [132,139,140]. NSCLC is one of the most common cancer types and a leading cause of cancer-related deaths worldwide [141]. In patients with EGFR-mutant NSCLC, tyrosine kinase inhibitors (TKIs) such as gefitinib, erlotinib, afatinib, and osimertinib have shown clinical efficacy. However, while many patients initially respond well to these targeted therapies, resistance typically develops within one year, limiting their long-term clinical benefit [142]. Multiple mechanisms contribute to resistance against osimertinib, including activation of alternative bypass signaling routes, aberrant downstream signaling, and histologic transformation. Among these, activation of the Akt pathway is frequently observed in EGFR-mutated NSCLCs with acquired resistance [132,143]. Among the three identified Akt isoforms (Akt1, Akt2, and Akt3), aberrant upregulation of Akt3 has been particularly implicated in supporting the survival of NSCLC cells once they develop resistance to osimertinib. Thus, TPDs against Akt3 could provide a new option to overcome this resistance. To address this, Xu et al. [132] developed a series of HyTDs designed to target Akt3. Through a virtual screening of an in-house compound collection, XTF-262 was identified as a novel Akt ligand. They subsequently designed a series of HyTDs by linking an adamantane group to XTF-262 through different linker moieties. Notably, compound 12l (Figure 2C) demonstrated notable potency and selectivity in a panel of NSCLC cell lines, achieving a DC_50_ against Akt3 of 13 nM and a maximum degradation efficiency of 88%, with minimal effects on Akt1/2. Thus, the development of 12l highlights the effectiveness of the HyT approach in targeting Akt3 to overcome acquired therapeutic resistance in NSCLC.

### 5.3. Enhancer of Zeste Homolog 2

Enhancer of Zeste Homolog 2 (EZH2) is a catalytic subunit of the polycomb repressive complex 2 (PRC2) that functions primarily as a transcriptional repressor, including the silencing of tumor suppressor genes. Its dysregulation accelerates cell proliferation and prolongs cell survival, promoting cancer development [144,145]. Mutations and/or altered expression of EZH2 are associated with many diseases, including melanoma, breast cancer, prostate cancer, lung cancer, liver cancer, psoriasis, hematological malignancies, and other disorders [146]. Thus, EZH2 has emerged as a high-impact molecular target in cancer. EZH2 is known to play a role in TNBC, which accounts for 15–20% of all breast cancer cases and remains the only subtype of breast cancer lacking molecularly targeted treatments [147]. Ma et al. [41] developed a first-in-class EZH2 selective degrader, MS1943 (Figure 2D). The authors discovered the EZH2-selective inhibitor C24, which exhibited more than 200× greater selectivity for EZH2 than for the closely related H3K27 methyltransferase EZH1. Docking studies of C24 with human PRC2 crystal structures revealed that its piperazine group of C24 is solvent-exposed. This orientation provided a suitable attachment point for introducing a hydrophobic moiety, including an adamantane group, via a linker, thereby converting C24 into MS1943. This novel HyTD was highly selective for EZH2 over EZH1 and other histone methyltransferases, with an IC_50_ of 120 nM against EZH2 compared with >10 μM against EZH1. MS1943 showed no activity against 45 additional protein kinases, including tyrosine and serine/threonine kinases, with less than 10% inhibition at 10 μM. Similarly, it showed less than 50% inhibition at 10 μM for 43 out of 45 tested G protein-coupled receptors (GPCRs), ion channels, and transporters. Notably, MS1943 reduced EZH2 protein levels in TNBC, lymphoma cell lines, and non-cancerous prostate cells, and intraperitoneal delivery of 150 mg/kg of MS1943 suppressed tumor growth in MDA-MB-468 TNBC cell line-derived xenografts.

Quantitative real-time PCR analyses revealed that MS1943 treatment in MDA-MB-468 TNBC cells induces upregulation of *Xbp1* and its downstream effectors, *Chop* and *BiP*, within 4 h, an effect sustained for at least 48 h. This prolonged induction suggests that EZH2 clearance leads to chronic ER stress and sustained overactivation of the UPR pathway, ultimately leading to apoptosis. Crucially, while C24 fails to alter the transcription of these stress-related genes, MS1943 drives their selective de-repression across multiple sensitive TNBC cell lines (MDA-MB-468, BT549, and HCC1187). In contrast, this transcriptional upregulation is completely absent in MS1943-insensitive MDA-MB-231 cells. Collectively, these findings indicate that MS1943 mediates its cytotoxic effects by exploiting ER stress and UPR induction in cells that depend on EZH2.

## 6. Limitations of Hydrophobic Tag Degraders

A major challenge for the field is that our understanding of how HyTDs degrade proteins remains in its early stages, making it difficult to predict whether a given POI can be successfully degraded using hydrophobic tagging [148]. In addition, HyTDs’ degradative potency is not as significant as that of PROTACs [149,150]. This is observed by Guo et al. [113], who conjugated an adamantane moiety to the KRAS-PDEδ inhibitor deltazinone, thereby generating HyTD 17c, which exhibited a DC_50_ of 11.4 μM in SW480 cells. Conversely, an analogous cereblon-based PROTAC (17f) previously synthesized by the same group using the same POI ligand achieved a DC_50_ of 3.6 μM in the same cell line [151]. This demonstrates that HyTDs require higher concentrations to maintain enough surface hydrophobicity to drive the POI toward degradation.

Compounding these potency challenges is the structural and biological unpredictability of the mechanism of action. While certain moieties can be exploited to favor distinct clearance routes, such as Boc_3_Arg, directing client proteins into the ubiquitin-independent proteasome system [81], a particular hydrophobic moiety does not guarantee a conserved degradation trajectory. For instance, despite both incorporating adamantane as the hydrophobic moiety, HyT-S7 directs its POI through the ALP [21], whereas YL2-HyT6 triggers canonical UPS clearance [24]. Due to differences in POI and linker properties, such as length and flexibility, the cell’s quality-control machinery engages each substrate uniquely. Even in systems where Boc_3_Arg clearly triggers UbInPD, multiple competing models exist regarding how the moiety presents the POI to the proteasome core [20], underscoring that the precise molecular mechanisms remain to be fully resolved. Consequently, the mechanism of action cannot be inferred from the hydrophobic moiety alone; each degrader requires rigorous experimental validation to elucidate its degradation pathway.

Lastly, off-target liabilities present a significant barrier to clinical translation. While Boc_3_Arg has been leveraged as a prototypical hydrophobic moiety, the free Boc_3_Arg moiety was shown to suppress cellular translation machinery by inhibiting the mTORC1 pathway [152]. This off-target limits the clinical use of Boc_3_Arg-based HyTDs [30]. Together, the off-target effects, mechanistic gaps, and lower potency profiles highlight that extensive chemical optimization remains necessary to bolster selectivity, potency, and enable reliable in vivo translation [153].

## 7. Conclusions

HyTDs provide several advantages over other targeted protein degradation strategies, such as PROTACs or MGDs, including (i) lower molecular weight, which often translates to improved cellular permeability; (ii) engaging diverse cellular clearance networks, including the UPS, UbInPD, ALP, and UPR, rather than relying on a narrow pool of specific E3 ligases; and (iii) straightforward design principles. However, pharmacological and mechanistic limitations must be resolved before HyTDs can achieve clinical translation. Most notably, unelucidated mechanisms of action, lower degradation potency compared to PROTACs, and off-target effects. Gaining a deeper, clearer insight into these precise mechanics is critical, as it will allow researchers to rationally optimize the molecules and fully exploit the cell’s endogenous degradation pathways. In addition to solving these mechanistic questions, future optimization must refine the POI ligand itself and maximize its binding affinity for the target protein. Ultimately, while HyTDs have already demonstrated their value as powerful tools in cancer research, further studies will be essential to fully unlock their greater potential in clinical research and therapy.

## Figures and Tables

**Table 1:** Reported HyTDs of Oncoproteins.

HyTD Name	Target Protein	POI Ligand	Linker	Hydrophobic Moiety	Degradation System	DC_50_	Year	Reference
SARD279	AR	RU59063	PEG	Adamantane	UPS	1 μM	2015	[22]
MS1943	EZH2	C24	Amide	Adamantane	UPR	12 nM (IC_50_)	2020	[41]
YL2-HyT6	SRC-1	YL2	PEG	Adamantane	UPS	5 μM	2021	[24]
17c	PDEδ	Deltazinone	Alkyl chain	Adamantane	ND ^a^	11.4 μM	2022	[113]
12l	Akt3	XTF-262	Alkyl chain	Adamantane	UPS	13 nM	2022	[132]
HyT-9	ALK	Alectinib derivative (A3)	Alkyl chain	Norbornene	UPS	134 nM	2023	[25]
SMD1	BRD4	JQ1	NASA	Adamantane	UPS & ALP	3 μM	2023	[117]
ARL-PLA-SS-PEG	AR	AR ligand (derived from enzalutamide)	PEG	PLA polymer	UPS & ALP	18.25 μM	2025	[133]

^a^ Not Determined.

## Data Availability

No new data were created or analyzed in this study. Data sharing is not applicable to this article.

## Abbreviations

The following abbreviations are used in this manuscript:

3-MA	3-methyladenine
ADT	Androgen Deprivation Therapy
Akt3	AKT Serine/Threonine Kinase 3
ALK	Anaplastic Lymphoma Kinase
ALP	Autophagy-Lysosome Pathway
AR	Androgen Receptor
ATF6	Activating Transcription Factor 6
Boc_3_Arg	Tert-Butyl Carbamate-Protected Arginine
BRD4	Bromodomain-Containing Protein 4
CHX	Cycloheximide
CMA	Chaperone-Mediated Autophagy
CQ	Chloroquine
CRPC	Castration-Resistance Prostate Cancer
DBP	DNA-Binding Protein
ER	Endoplasmic Reticulum
EZH2	Enhancer of Zeste Homolog 2
Fur	Thiobenzofurazan
GPCRs	G protein-Coupled Receptors
GST	Glutathione-S-transferase
HBC	Hepatitis B Virus Core
HBV	Hepatitis B Virus
HNSCC	Head and Neck Squamous Cell Carcinoma
HyT	Hydrophobic Tag
HyTD	Hydrophobic Tag Degrader
IDPs	Intrinsically Disordered Proteins
IDRs	Intrinsically Disordered Regions
IRE1α	Inositol-Requiring Enzyme 1α
LACHT	Ligand-Assisted Covalent Hydrophobic Tagging
MGD	Molecular Glue Degrader
NASA	N-Acyl-N-alkyl Sulfonamide
NEF	Nucleotide Exchange Factor
NSCLC	Non-Small Cell Lung Cancer
ODC	Ornithine Decarboxylase
PARP1	Poly (ADP-ribose) Polymerase 1
PDEδ	Phosphodiesterase 6δ
PEG	Polyethylene Glycol
PERK	Protein Kinase R-like Endoplasmic Reticulum Kinase
PLA	Polylactic Acid
POI	Protein of Interest
PRC2	Polycomb Repressive Complex 2
PROTAC	Proteolysis-Targeting Chimera
RBP	RNA-Binding Protein
SAR	Structure–Activity Relationship
SMDs	Small-Molecule Degraders
SMIs	Small-Molecule Inhibitors
SRC-1	Steroid Receptor Coactivator-1
TKIs	Tyrosine Kinase Inhibitors
TNBC	Triple-Negative Breast Cancer
TPD	Target Protein Degradation
TS	Thymidylate Synthase
UbInPD	Ubiquitin-Independent Proteasome Degradation
UPR	Unfolded Protein Response
UPS	Ubiquitin-Proteasome System
